# Nanostructure Mediated Piezoelectric Effect of Tetragonal BaTiO_3_ Coatings on Bone Mesenchymal Stem Cell Shape and Osteogenic Differentiation

**DOI:** 10.3390/ijms24044051

**Published:** 2023-02-17

**Authors:** Yafei Zheng, Lingzhou Zhao, Ying Li, Xinyuan Zhang, Wei Zhang, Jing Wang, Lipeng Liu, Weikang An, Hua Jiao, Chufan Ma

**Affiliations:** 1 State Key Laboratory of Military Stomatology & National Clinical Research Center for Oral Diseases & Shaanxi Key Laboratory of Stomatology, Department of Prosthodontics, School of Stomatology, The Fourth Military Medical University, Xi’an 710032, China; 2 Air Force Medical Center, The Fourth Military Medical University, 30 Fucheng Road, Beijing 100089, China; 3 School of Materials Science and Engineering, Xi’an University of Technology, Xi’an 710048, China

**Keywords:** Barium titanate, tetragonal, mesenchymal stem cells, cell shape, osteogenic differentiation

## Abstract

In recent years, porous titanium (Ti) scaffolds with BaTiO_3_ coatings have been designed to promote bone regeneration. However, the phase transitions of BaTiO_3_ have been understudied, and their coatings have yielded low effective piezoelectric coefficients (EPCs < 1 pm/V). In addition, piezoelectric nanomaterials bring many advantages in eliciting cell-specific responses. However, no study has attempted to design a nanostructured BaTiO_3_ coating with high EPCs. Herein, nanoparticulate tetragonal phase BaTiO_3_ coatings with cube-like nanoparticles but different effective piezoelectric coefficients were fabricated via anodization combining two hydrothermal processes. The effects of nanostructure-mediated piezoelectricity on the spreading, proliferation, and osteogenic differentiation of human jaw bone marrow mesenchymal stem cells (hJBMSCs) were explored. We found that the nanostructured tetragonal BaTiO_3_ coatings exhibited good biocompatibility and an EPC-dependent inhibitory effect on hJBMSC proliferation. The nanostructured tetragonal BaTiO_3_ coatings of relatively smaller EPCs (<10 pm/V) exhibited hJBMSC elongation and reorientation, broad lamellipodia extension, strong intercellular connection and osteogenic differentiation enhancement. Overall, the improved hJBMSC characteristics make the nanostructured tetragonal BaTiO_3_ coatings promising for application on implant surfaces to promote osseointegration.

## 1. Introduction

Titanium (Ti) and its alloys are commonly used as bone and dental implants based on their good biocompatibility and excellent mechanical properties. However, they are essentially bio-inert, leading to delayed and frequently unsatisfactory osseointegration. The past decade has witnessed unprecedented research progress with the advent of surface coating technology that can endow Ti implants with extra bioactivity while maintaining Ti’s good biocompatibility and mechanical properties [1]. Bone-driven biological coatings fabricated by stimulating the physiological bone microenvironment may generate bone implants with enhanced osseointegration and better clinical performance. Unlike biological molecules and chemicals, surface physical cues with adjustable properties have become a research hotspot since they can be applied in a timely and localized manner [2]. The natural bone extracellular matrix (ECM) contains nanoscale cues that are widely believed to play crucial roles in bone turnover. Inspired by this, Ti with myriad nano-textured coatings has been developed to enhance the bioactivity of Ti implants [1].

Endogenous electric fields exist in the body and are essential for tissue development, remodeling, and repairment [3]. Natural bone tissues exhibit electromechanical coupling [4], which has long been thought to be attributed to the piezoelectricity of collagen [5]. A recent study demonstrated that most of the electromechanical response of bone comes from the flexoelectricity of bone minerals rather than collagen [6]. Besides, ionic streaming potentials contribute to the electromechanical properties of wet bone. These have encouraged research on the biological effect of external electric fields/currents, with an increasing body of evidence of their effects on inducing osteogenesis [7]. Clinically, external electric devices have been used successfully to treat bone fractures since the 1970s [8]. However, the clinical application of such large-sized extracorporeal equipment is limited by the need for professional equipment and complex implementation. Compared to external macroscopic electric fields, those built-in on the implant surface can provide more clinical convenience and may be better perceived by the cell surface receptors to regulate their functions. Accordingly, pre-charged biomaterial surfaces with polarized ferroelectrics have been developed to influence cell functions and osseointegration [9,10,11]. Current evidence suggests that positive surface charges promote the spreading and enhance osteogenic differentiation of mesenchymal stem cells compared to the negative and neutral charges [9,10]. Besides, nanocomposite membranes with negative potential have been observed to enhance bone regeneration [11]. Indeed, it is highly likely that the surface charges have a limited effective period since they will decay over time [11]. The electromechanical coupling nature of bone may be simulated by piezoelectric material coatings, which can generate surface electric charges in response to mechanical stress or deformation, to provide localized sustained and dynamic mechanical-responsive electrical stimuli [12]. Mechanical stress or deformation can originate from biological activities such as cell spread, traction and migration, body motion, and external stimulators, especially ultrasound [13]. Barium titanate (BaTiO_3_), discovered to be piezoelectric early in 1946 [14], has good biocompatibility and has attracted much attention in the field of biomedical science [15,16,17]. As a perovskite material, BaTiO_3_ has five crystalline phases, among which the tetragonal phase, an asymmetrical structure, has attracted significant interest for practical applications due to its excellent piezoelectric effect [18]. Piezoelectric nanomaterials are advantageous in eliciting cell-specific responses due to their nano size, surface nanostructure, high surface-area-to-volume ratio, and high surface energy compared to their bulk or microscale counterparts [13].

In recent years, much emphasis has been placed on building BaTiO_3_ coatings on Ti porous scaffolds to promote bone regeneration [19,20,21,22]. Unfortunately, most studies did not explore the phase transition of the BaTiO_3_ coatings, and none developed a nanostructured BaTiO_3_ coating [19,20,21,22]. It is well-established that bulk BaTiO_3_ can be easily manipulated to exhibit tetragonal ferroelectricity at room temperature, which is gradually reduced with decreasing particle size and disappears below a certain threshold (in the range of 10–100 nm) [23]. Hence, it remains challenging to fabricate nanostructured BaTiO_3_ coating on Ti that exhibits tetragonal ferroelectricity, nor is the combined effect of nanostructured tetragonal phase BaTiO_3_ with piezoelectric properties as Ti implant coatings on the functions of bone mesenchymal stem cells (MSCs) clear. In this paper, tetragonal BaTiO_3_ nanoparticulate coatings with effective piezoelectric coefficients of 10–180 pm/V were developed on Ti by anodization combing two hydrothermal processes. Importantly, their effects on the cell morphology and osteogenic differentiation of human jaw bone marrow mesenchymal stem cells (hJBMSCs) were studied.

## 2. Results and Discussion

**Figure 1:**
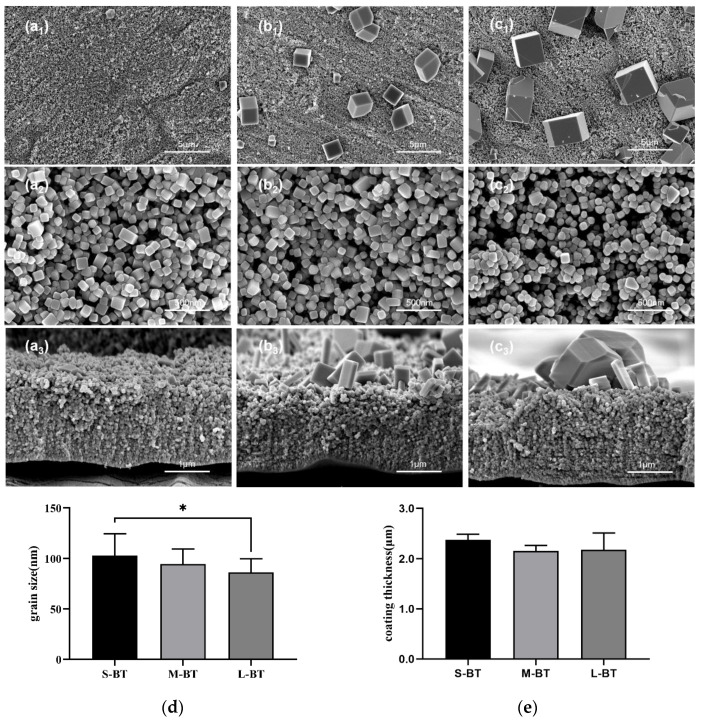
SEM micrographs of surface morphology of low magnification (**a1**–**c1**) and high magnification (**a2**–**c2**), and corresponding cross-sections (**a3**–**c3**) of the S-BT (**a1**–**a3**), M-BT (**b1**–**b3**), and L-BT (**c1**–**c3**). (**d**) Grain size of the cube-like nanoparticles on the three Ti surfaces. (**e**) Coating thickness measured from the cross-sections. Data are presented as mean ± SD, * *p* < 0.05.

Figure 1 shows the SEM micrographs of surface morphology and corresponding cross-sections of S-BT(Coating with small cube-like BaTiO_3_ particles on the surface) (Figure 1a1,a2,a3), M-BT(Coating with middle cube-like BaTiO_3_ particles on the surface) (Figure 1b1,b2,b3), and L-BT(Coating with large cube-like BaTiO_3_ particles on the surface)(Figure 1c1,c2,c3), which correspond to 12, 24, and 36 h during the secondary hydrothermal reaction, respectively. Evenly distributed cube-like nanoparticles were observed on the three kinds of Ti samples, as shown in the higher magnification surface morphology SEM micrographs (Figure 1a2,b2,c2). On S-BT, the size of the nanoparticles was 103 nm, and a decrease in size was observed with time (95 and 86 nm for M-BT and L-BT, respectively, Figure 1d). The edges of cube-like nanoparticles were relatively sharp on S-BT, which became smoother with time (Figure 1a2,b2,c2). The surface morphology SEM micrographs at low magnification (Figure 1a1,b1,c1) showed that sparsely distributed cube-like microparticles with sharp edges gradually emerged on M-BT and L-BT, and their size was positively correlated to the reaction time. The mean microparticle size was 1.86 and 3.03 μm for M-BT and L-BT. Cross-sectional observation of the coatings (Figure 1a3,b3,c3) showed that the grain size decreased gradually from top to bottom. The coating thickness values of M-BT and L-BT were slightly smaller than S-BT (Figure 1e).

**Figure 2:**
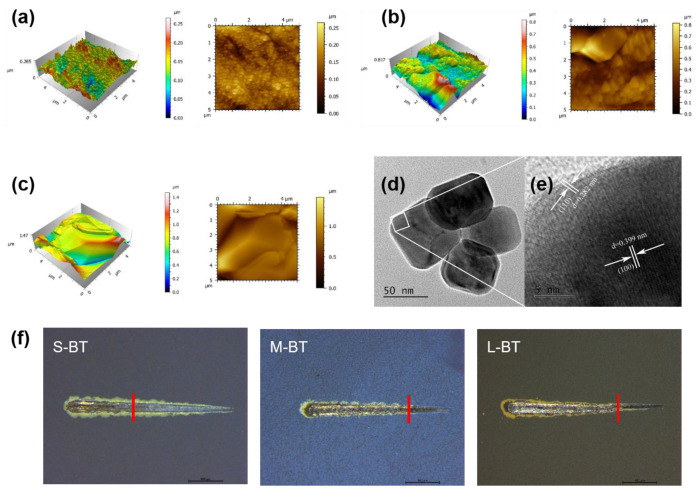
AFM 3D reconstruction (left) and roughness (right) of the Ti surfaces: (**a**) S-BT, (**b**) M-BT and (**c**) L-BT. TEM image of the cube-like nanoparticles from S-BT (**d**) and HRTEM image (**e**) obtained from the area outlined by the bright square in (**d**). (**f**) The images of the scratch test of the S-BT, M-BT, and L-BT. The red line represents the critical load point.

Figure 2a–c shows the AFM 3D reconstruction and roughness of the Ti surfaces. Overall, the surface morphology of each group was consistent with the SEM results. For the S-BT group, the Sa (arithmetic mean height) value was 0.0239 μm, and the Sq (root mean square height) value was 0.0307 μm, which means that the surface coating of the S-BT group has a typical nanomorphology. Unfortunately, due to the sparse distribution of larger particles on the surface of M-BT and L-BT and the limited scanning range of the AFM probe, the surface roughness data obtained had no practical significance and could not be analyzed. The cube-like nanoparticles on the Ti samples were scraped off for TEM inspection. No significant difference was observed for nanoparticles from different Ti samples. The representative TEM and S-BT images are shown in Figure 2d,e. The internal structure of the nanoparticles was compact without defects. HRTEM image (Figure 2e) obtained from the area outlined by the bright square in Figure 2e showed that the nanoparticles have good crystallinity, and the lattice stripes of the small particles were clear. The labeled spacings of 0.282 and 0.399 nm were consistent with the (110) and (100) crystal plane spacings of BaTiO_3_, respectively. The EDS data (Figure S1) further showed that BaTiO_3_ particle was successfully immobilized on the surface of the Ti since Barium was observed in the EDS data.

It is well-established that the strong bonding strength between the implant and the coating is essential. Otherwise, the coating may peel off during the implantation process, affecting the osseointegration of the implant. Figure 2f shows the typical images of scratch tracks for different samples during the scratch test. Overall, the coating was deformed and destroyed due to the large strain energy generated around the diamond indenter during the scratch test. The load at which the coating is completely stripped from the substrate is called the critical load (Lc) [24,25]. Under the same experimental load and scratch length, the later the stripping point appears, the stronger the coating adhesion is. In our study, the stripping point of the S-BT appeared later than for M-BT and L-BT, indicating that the bonding strength of S-BT was better than M-BT and L-BT. Since the bond strength can be calculated by the ratio of the critical load to the surface area of the scratch tip, we calculated that the bond strength of S-BT, M-BT and L-BT groups is 140, 70, and 56.6 Mpa, respectively. Interestingly, the bonding strength of S-BT was superior to hydroxyapatite (HA) on titanium substrates by various methods in previous reports [26]. Meanwhile, we acknowledge that the bonding strength values obtained from scratch tests were difficult to compare across different studies due to the critical load loading method, instrument type, and different coating/substrate surface influence. Further “pull-out” tests in animal experiments [27] are warranted to confirm the sufficiency of coating strength to withstand the forces bearing during surgery and functioning.

**Figure 3:**
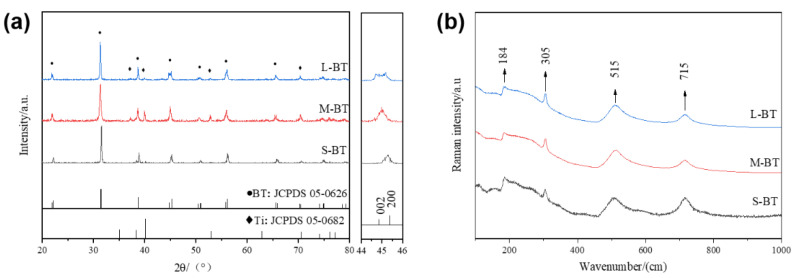
Sample characterization: (**a**) XRD patterns and (**b**) Raman spectra.

Figure 3 shows the conventional XRD patterns and the Raman spectra of the Ti specimens. The XRD patterns of all samples (Figure 3a) corresponded to typical BaTiO_3_ peaks (JCPDS NO. 05-0626). More significant splitting of the Bragg reflection angle 2*θ* located at ∼45° and indexed as (002)/(200) was observed from 12 h to 36 h. When the lattice structure of BaTiO_3_ changed from cubic (centrosymmetric) to tetragonal phase (non-centrosymmetric), the lattice was distorted, leading to a lengthened c axis, shortened a axis, and c/a value >1. The calculated c/a ratios were 1.0038, 1.0065, 1.0078 for S-BT, M-BT, and L-BT, respectively, which indicate that BaTiO_3_ on Ti samples were in the tetragonal phase. Raman scattering is a powerful tool for investigating the phase transition of BaTiO_3_. The sharp peaks in the Raman spectra at 185, 305, 515, and 715 cm^−1^ and the absence of a peak at 190 cm^−1^ (Figure 3b) also indicate the formation of the tetragonal phase BaTiO_3_ on the three Ti samples [18]. Overall, it can be concluded that unique BaTiO_3_ cube-like nanoparticulated coatings exhibiting a tetragonal phase on Ti substrate were successfully developed. This is the first report on nanostructured tetragonal BaTiO_3_ coating on Ti implant, which is of great significance for studying the combined biological effect of nano dimension and piezoelectricity.

**Figure 4:**
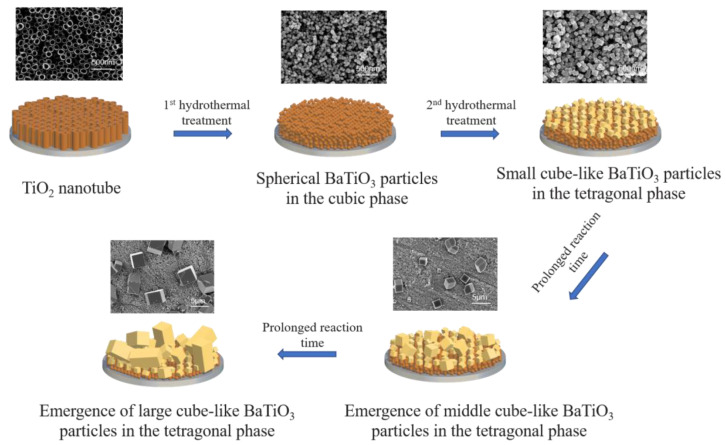
Schematic illustration for the formation process of the tetragonal BaTiO_3_ nanoparticulate coatings.

Figure 4 depicts the conversion process from the TiO_2_ nanotube array to large BaTiO_3_ nanoparticles. Even though the conversion of titanium dioxide nanotubes to BaTiO_3_ has previously been described in the literature [28], the mechanism seems different in the present study. The complete disappearance of the nanotube structure suggested that the formation of spherical BaTiO_3_ particles during the first hydrothermal reaction was not achieved by in situ transformation but by a dissolution–precipitation mechanism, which can be attributed to high temperature (230 °C), high barium concentration and strong alkali concentration that are the key parameters for nucleation and crystal growth of BaTiO_3_ [29]. During the secondary hydrothermal process, the BaTiO_3_ morphology changed from spherical to cube-like and from cubic phase to tetragonal phase, with the tetragonal characteristics becoming more apparent with time due to the Ostwald ripening process. With time, the smaller particles dissolved, diffused and redeposited on the surface of larger particles, leading to the emergence of microparticles with increased size [30]. Importantly, we observed decreased nanoparticle size, smoothening edges of the nanoparticles, and slightly decreased coating thickness with the reaction time (Figure 1). Spanier et al. [31] reported that adsorption of the hydroxyl group was beneficial to the stability of the ferroelectricity of BaTiO_3_. Hongo et al. [32] established a model to confirm that the appropriate concentration of hydroxyl incorporation is conducive to the simultaneous contraction of the a and b axes of the lattice and elongation along the c axis, important for the stability of the tetragonal phase structure. Hence, the hydroxyl groups generally formed on the material’s surface during alkali hydrothermal treatment play an important role during the transformation from the cubic phase to the tetragonal phase during the second hydrothermal process.

**Figure 5:**
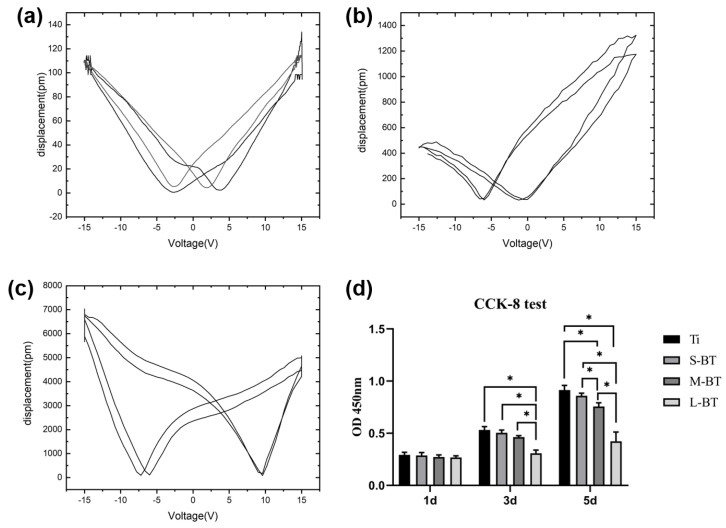
The hysteresis loop and the displacement amplitude of the PFM switching of the BaTiO_3_ coatings on Ti: (**a**) S-BT, (**b**) M-BT, and (**c**) L-BT. (**d**) Cell viability/proliferation of hJBMSCs after incubation for one, three, and five days on different Ti samples. Data are presented as mean ± SD, * *p* < 0.05.

Piezoresponse force microscopy is often used to measure the ferroelectricity and piezoelectricity of nanomaterials via an indirect piezoelectric effect, in which the material deforms under the influence of an applied electric field [15]. Figure 5a–c shows the hysteresis loop and the displacement amplitude of the PFM switching of the BaTiO_3_ coatings on Ti. A typical “butterfly” loop was observed on the displacement versus voltage diagram for each group, typical of ferroelectric materials [33]. For the indirect piezoelectric effect, the expression of piezoelectric strain is ε = dE, where ε, d, and E are strain, piezoelectric strain coefficient, and electric field, respectively [15]. Accordingly, the effective piezoelectric coefficient d_33_ was calculated based on the “butterfly” loop in Figure 5, yielding values of 10, 60, and 180 pm/V for S-BT, M-BT, and L-BT, respectively. The gradual increase in piezoelectric coefficient value was ascribed to the emergence of microparticles of increased size. It has been reported that the piezoelectric coefficient of bulk BaTiO_3_ fabricated by conventional sintering using chemical powder is about 190 pC/N [34]. We found that the piezoelectric coefficient of L-BT was comparable to bulk BaTiO_3_. It has been reported that the piezoelectric coefficient of dry bone is 0.7 pC/N [35], while that of wet bone is about 8 pC/N [36]. Hence, S-BT can reportedly mimic the piezoelectric coefficient of bone, and M-BT and L-BT can yield the biological effect of supraphysiological ones. In this respect, the BaTiO_3_ coatings fabricated by Liu et al. [20] and Cai et al. [22] showed piezoelectric coefficients of 0.7 pC/N [20] and 0.42 pC/N [22], respectively, which are similar to dry bone. The coatings developed in the present study generated significantly higher piezoelectric coefficients than reported in the literature in a context where achieving high tetragonality and piezoelectricity at the nanoscale level is challenging [23].

**Figure 6:**
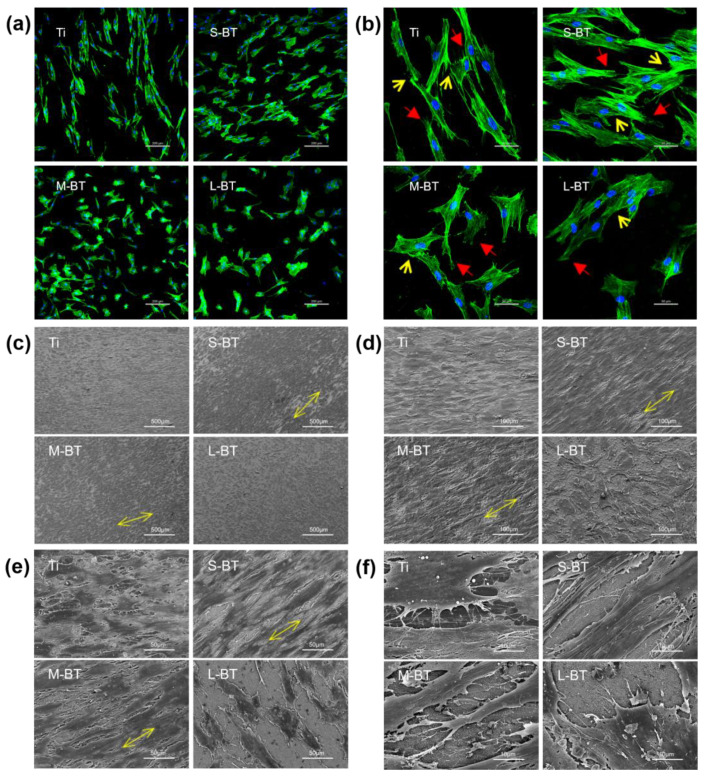
Cell morphology and geometry by immunofluorescence (**a**,**b**) and SEM (**c**–**f**). Fluorescent staining of cell actin skeleton after culturing for three days on all Ti surfaces (staining: blue-nuclei, green-actin): (**a**) lower magnification, and (**b**) higher magnification. The red arrows indicate the cell protrusions, and the yellow arrows point to the intercellular connections. SEM micrographs of hJBMSC morphology on different Ti samples after incubation for five days. The magnification increases gradually from (**c**–**f**). The yellow arrows show the direction of the cell orientation.

**Figure 7:**
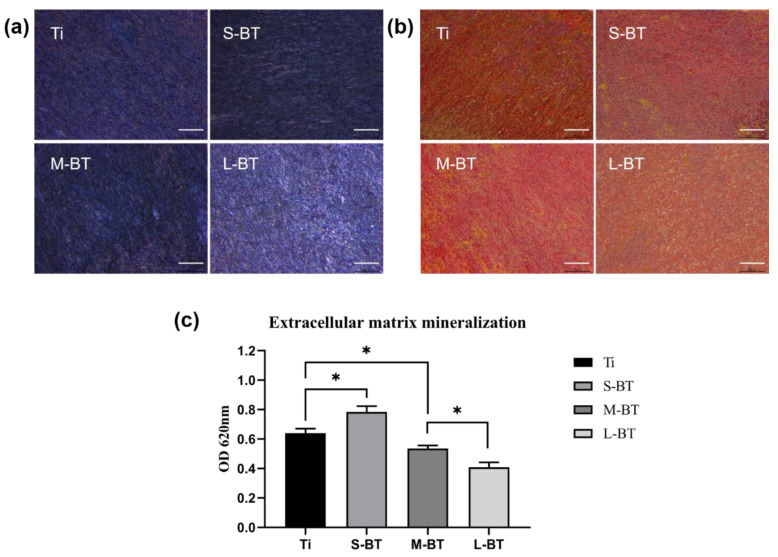
The osteogenic differentiation of hJBMSCs on the Ti samples under dynamic pressurized conditions. (**a**) alkaline phosphatase staining, (**b**) Alizarin Red staining, and (**c**) semi-quantitative analysis of Alizarin Red staining. Bars = 500 μm. Data are presented as mean ± SD, * *p* < 0.05.

Electrical stimulation has been demonstrated to show a dose-dependent effect on the behavior of MSCs [37,38]. Hence it is widely thought that the biological effect of piezoelectric coatings yields a piezoelectric coefficient-dependent effect. However, little attention has been paid to the piezoelectric coefficient effect on cell functions. The combined effect of the nanostructure and different piezoelectric coefficients on the functions of hJBMSCs was explored, and BaTiO_3_ was found to be biocompatible [1]. The hJBMSCs used in this experiment conform to the characteristics of MSCs, which can be used in the later experiment (Figure S2). Comparable cell viability was observed after one day of culture. The gradual proliferation of hJBMSCs on all Ti surfaces (Figure 5d) and good cell attachment and spreading after three and five days of culture (Figure 6 and Figure 7) on all Ti surfaces validated the good biocompatibility of the BaTiO_3_ nanostructured coatings. Good biocompatibility means that the material implanted in the body will not cause toxic effects on cells. After three and five days of culture, after pure titanium was taken as the control group, M-BT showed slight growth inhibition while L-BT inhibited cell growth. Current evidence suggests that piezoelectric biomaterial (piezoelectric coefficients of 10 pC/N) exhibits an inhibitory effect on MSCs growth [38]. Meanwhile, the BaTiO_3_ coatings with piezoelectric coefficients of 0.7 pC/N [20] and 0.42 pC/N [22] yielded a stimulatory effect on cell proliferation. Thus, it may be concluded that the effect of the piezoelectric coefficient on cell proliferation is dose-related, with a smaller piezoelectric coefficient (0.4–0.7 pC/N) enhancing cell proliferation and a larger piezoelectric coefficient (≥10 pC/N) inhibiting cell growth.

Cell morphology and geometry influence cellular processes such as stem cell differentiation, which the biomaterial surface nanotopography can modulate. Indirect mechanotransduction of integrin dependent signal pathways and direct mechanotransduction of gene expression originated from cell nucleus distortion by force transferred via the cytoskeleton represent the mechanisms by which biomaterials steer stem cell fate [39,40]. No significant difference in the cell morphology was observed from the lower magnification images of cell skeleton staining after three days of culture (Figure 6a). Relatively fewer cells were found on L-BT compared to the other groups, which is in accordance with the cell proliferation assay results (Figure 5d). Observation at higher magnification (Figure 6b) showed that the cells on Ti control tended to elongate many thin filopodia (red arrows) and exhibited relatively thin intercellular connections (yellow arrows). However, on the three BaTiO_3_ coatings, broad lamellipodia (red arrows) with broad and tight intercellular connections (yellow arrows) were mainly observed instead of thin filopodia. Filopodia are thin extensions at the cellular periphery responsible for cell migration [41], while lamellipodia are broad sheet-like cellular actin structures playing roles in cell motility, directional migration, cell polarity maintenance and environmental probing [42]. Besides cell-biomaterial interaction, cell-cell communication is also important for cell functions, which have been reported to be modulated by biomaterials [43]. Cell skeleton staining indicated that nanostructure and static electrical stimulation promoted arrangement and cell adhesion. Meanwhile, we also obversed hJBMSC morphology through SEM.

Cell geometry was further studied by SEM after five days of culture (Figure 6c–f). Observation at lower magnifications showed that on Ti and L-BT (Figure 6c,d), the cells adopted a polygonal cell shape with a random distribution of orientation and smaller abundance on L-BT. However, S-BT and M-BT induced the elongation of the cells and their reorientation in the same direction (indicated by the yellow arrows). The cells nearly reached confluence on Ti, S-BT, and M-BT after five days of culture, while those on L-BT only showed a nearly 50% coverage of the Ti surface. Further details are shown on the higher magnification SEM images in Figure 6e,f. The ventral-dorsal thickness of the cells was much larger on the three BaTiO_3_ coatings than on the Ti control, making the cells on the BaTiO_3_ coatings more stereoscopic. Long and strong lamellipodia were commonly seen on S-BT and M-BT, while short and thin filopodia were more prevalent on Ti. Accordingly, it is widely thought that S-BT and M-BT have more extensive intercellular connections. To conclude, BaTiO_3_ coatings of 10 and 60 pm/V lead to elongation, reorientation, lamellipodia stretching, and solid intercellular connection of hJBMSCs.

Figure 7 shows the results of the osteogenic activity evaluation under dynamic pressurized conditions in vitro. After seven days of osteogenic induction, ALP activity (a marker for early osteogenic differentiation) was most prominent on different Ti samples of S-BT, followed by M-BT, Ti and L-BT (Figure 7a). The ECM mineral deposition visualized by Alizarin Red staining was most prominent in S-BT, followed by Ti, M-BT and L-BT (Figure 7b,c). The results demonstrate that in the presence of nanostructure, BaTiO_3_ coating of 10 pm/V exhibits a stimulatory effect on hJBMSC osteogenic differentiation. Above result can be explained that the coatings provide a suitable surface topography and generate an endogenous electric field by mechanical deformation, which can promote hJBMSC cell adhesion and osteogenic differentiation.

## 3. Materials and Methods

### 3.1. Coating Preparation

Ti disk (10 mm in diameter and 1.5 mm in thickness) treatment and anodization were performed according to a protocol described in the literature [44]. In brief, the nanotube array coatings were fabricated on Ti in the ethylene glycol (EG, Macklin Biochemical, Shanghai, China) electrolyte containing 0.5 wt% NH_4_F (Macklin Biochemical, Shanghai, China), 5 vol% CH_3_OH (Fuyu Chemical, Tianjin, China), and 5 vol% H_2_O at 40 V for 1 h. Then the Ti samples with nanotube array coatings were placed in the PPL-Teflon-lined stainless-steel reactor containing a mixture of 12.5 M NaOH (Macklin Biochemical, Shanghai, China) solution and 1 M BaCl_2_ (Macklin Biochemical, Shanghai, China) solution with a ratio of 2:1, filled to 50% of its capacity, to construct BaTiO_3_ film on Ti substrate. The reactor was kept at 230 °C for 24 h as the first hydrothermal reaction. Next, the BaTiO_3_ film was introduced to 3.33 M NaOH solution for a secondary hydrothermal reaction at 230 °C for 12, 24, and 36 h to produce BaTiO_3_ cube-like nanoparticulate films. The secondary hydrothermal reaction products were named according to their morphologies under SEM.

### 3.2. Sample Characterization

Surface morphology and coating cross-section of the Ti samples were observed on field emission scanning electron microscopy (FE-SEM 4800; Hitachi, Tokyo, Japan) with an acceleration voltage of 5 keV. The Ti surface was coated with gold for optimal imaging. Surface roughness (Ra) of the specimens was analyzed using atomic force microscopy (AFM; Agilent Technologies, Santa Clara, CA, USA) with a scanning area of 5 μm × 5 μm at a scanning rate of 10 μm/s. The particles on Ti samples were scraped off and dispersed by ultrasonics in anhydrous ethanol. Transmission electron microscopy (TEM) and high-resolution TEM (HRTEM) micrographs of the nanoparticles scraped off from the Ti samples were taken on Transmission Electron Microscopes (HITACHI H-800 and JEOL 2010, Tokyo, Japan) at an accelerating voltage of 200 kV. Coating adhesion to substrates was evaluated using a scratch tester (WS-2005, CAS, Lanzhou, China) with a 0.2 mm tip radius diamond indenter, with an experimental load range of 0–30 N, scratch speed of 3 mm/min, and scratch length of 3 mm. The images were acquired with an optical microscope (Leica, Wetzlar, Germany). X-ray powder diffraction (XRD) patterns of the products were obtained on a Japan Rigaku D/Max-IIIC diffractometer at a voltage of 60 kV and a current of 80 mA with Cu Kα radiation (λ = 1.5406 A), employing a scanning rate of 8°/min in 2*θ* ranging from 20° to 80°. Raman spectrum was evaluated using a micro-Raman spectrometer (HR800; HORIBA Jobin Yvon, Paris, France) with a laser wavelength of 532 nm and exposure time of 80 s to acquire the reference peak (r_100_ cm^−1^) and reaction peak (r_1000_ cm^−1^) of the coatings. The piezoelectric properties of three BaTiO_3_ coatings were characterized by piezoresponse force microscopy (PFM; Nanoscope V Multimode 8, Bruker, Saarbrücken, Germany). The experiments were conducted under environmental conditions (temperature 25 °C, relative humidity 25%) using the same PFM probe.

### 3.3. Cell Culture and Identification

The hJBMSCs [45] (Research and Development Center for Tissue Engineering, Fourth Military Medical University) were grown in α minimum essential medium (α-MEM, Hyclone, South Logan, UT, USA) supplemented with 10% fetal bovine serum (FBS, EVERY GREEN, TIANHANG, Huzhou, China) and 1% Penicillin/streptomycin (Gibico, Carlsbad, CA, USA) at 37 °C in a humidified 5% CO_2_ atmosphere. The cells were passaged when the culture reached 80% confluence, and the experiments were performed at passage 4. Cell identification was performed as previously described [45,46]. Briefly, the abilities of plastic adherence, osteogenic differentiation, and adipogenic differentiation were studied to verify the MSC characteristics. Surface antigen (Ag) expressions were detected by flow cytometry to ensure that the cells were not confounded by other cells.

### 3.4. Sample Sterilization and Cell Seeding

Given that BaTiO_3_ undergoes a transition from a ferroelectric tetragonal phase to a paraelectric cubic phase when heating above 120 °C [23], the Ti samples are soaked in 75% medical alcohol (LIRCON, Shandong, China) for 6 h for sterilization instead of using autoclaving. Then the samples were exposed to ultraviolet irradiation of 2 h to achieve full sterilization. Finally, the samples were soaked in sterile phosphate buffer saline (PBS, Sigma-Aldrich, St. Louis, MO, USA) for 6 h to remove possible residual alkali ions before use. The Ti samples were mounted in 24 well plates (Corning, Shanghai, China). 2 × 10^4^ cells were suspended in 1 mL α-MEM with 10% FBS, and 1% Penicillin/streptomycin was seeded onto each Ti sample. The Ti samples were cultured at 37 °C in a humidified 5% CO_2_ atmosphere.

### 3.5. Cell Viability

Cell viability on the Ti samples was investigated using a Cell Counting Kit-8 assay (CCK-8; Dojindo, Kumamoto, Japan). The detection time points were one, three, and five days after cell seeding. Briefly, the Ti samples with cells were rinsed with PBS and transferred to a new 24-well plate. Then 300 mL of the a-MEM medium and 30 mL of the CCK-8 solution were added to each sample and incubated at 37 °C for 2 h. The absorbance was measured at 450 nm.

### 3.6. Cell Skeleton Staining

After the cells were cultured on each sample for three days, the cytoskeleton was stained with phalloidin (100 nM Acti-stainTM488, AmyJet Scientific, Wuhan, China) at room temperature for 30 min; then the nucleus was stained with DAPI-containing anti-fluorescence attenuation mounting medium (VECTASHIELD Antifade Mounting Medium, Annoron Biotechnology, Beijing, China). The stained specimens were stored in a dark box. Images were acquired using a Nikon A1 confocal microscope (Nikon, Tokyo, Japan).

### 3.7. Cell Shape Observation by SEM

On day 5, the cells on the Ti samples were washed with PBS, fixed with 2.5% glutaraldehyde, dehydrated with gradient ethanol, freeze-dried, and then observed under SEM after gold spraying.

### 3.8. Cell Osteogenic Differentiation

The osteogenic differentiation capacity of hJBMSCs on the Ti samples was detected under dynamic pressurized conditions in vitro. The culture media were supplemented with osteogenic induction solution (10 nM dexamethasone (Sigma, MO, USA), 50 mM ascorbate-2-phosphate (Sigma), 2 mM β-glycerophosphate (Sigma) and 10 nM 1,25-dihydroxyvitamin D3 (Sigma)). In this experiment, a pressurizing device was used, which was custom-made in a previous study [47]. Dynamic periodic pressure loading (dynamic pressure 90 kPa, frequency 0.1 Hz, and once a day for 1 h each time) was conducted through pressure conversion. After seven days of culture, the osteogenic cell differentiation was assessed.

#### 3.8.1. Alkaline Phosphatase Staining

For alkaline phosphatase staining, on day seven, the cells were washed with PBS, fixed in 3% glutaraldehyde for 30 min, and then stained with BCIP/NBT ALP kit (LEAGENE, Beijing, China). The images were acquired with an optical microscope (Leica, Germany).

#### 3.8.2. Alizarin Red Staining

For Alizarin Red staining, after washing with PBS and fixing, the cells were stained using 40 mM Alizarin Red (pH 4.2, Solarbio, Beijing, China) to assess mineralization. The unbound stain was washed with distilled water before the images were taken. The images were conducted with an optical microscope. Then 10% hexadecylpyridine chloride (Acros, China) eluent was added (1 mL/well) to measure the absorbance value at 620 nm wavelength for semiquantitative analysis.

### 3.9. Statistical Analysis

All data were statistically analyzed with the SPSS 22.0 software package (SPSS Inc., Chicago, IL, USA) and presented as the mean ± SD for each group. Comparisons were made at each time point using a one-way ANOVA. A *p*-value < 0.05 was statistically significant.

## 4. Conclusions

In the present study, nanostructured tetragonal BaTiO_3_ coatings with evenly distributed cube-like nanoparticles of about 90–100 nm in size of different effective piezoelectric coefficients of 10–180 pm/V were successfully fabricated through a novel method of anodization combing two hydrothermal processes. The coatings displayed good biocompatibility but yielded a piezoelectric coefficient-dependent inhibitory effect on hJBMSC proliferation. In vitro studies, including the CCK 8 assay, cell skeleton staining, osteogenic differentiation, alkaline phosphatase staining and alizarin red staining, suggested that the relatively smaller EPC of < 10 pm/V was associated with hJBMSC elongation and reorientation, broad lamellipodia extension, strong intercellular connections, and osteogenic differentiation, highlighting it has huge prospects for bone implant surface application to promote osseointegration.

## Data Availability

The raw data supporting the conclusion of this article will be made available by the authors, without undue reservation.

## Supplementary Materials

The supporting information can be downloaded at: https://www.mdpi.com/article/10.3390/ijms24044051/s1.
