# Supplementary material for: Nanostructure Mediated Piezoelectric Effect of Tetragonal BaTiO3 Coatings on Bone Mesenchymal Stem Cell Shape and Osteogenic Differentiation
Source: Int J Mol Sci. 2023 Feb 17;24(4):4051. doi: 10.3390/ijms24044051 (PMC9961896; doi:10.3390/ijms24044051)
Supplement: Supplementary file 1 [file ijms-24-04051-s001.zip › ijms-2152853-supplementary.pdf]

Supplementary File

The EDS data (Figure S1) further showed that BaTiO3 particle was successfully immobilized on the surface of the Ti because Barium was observed in the EDS data.

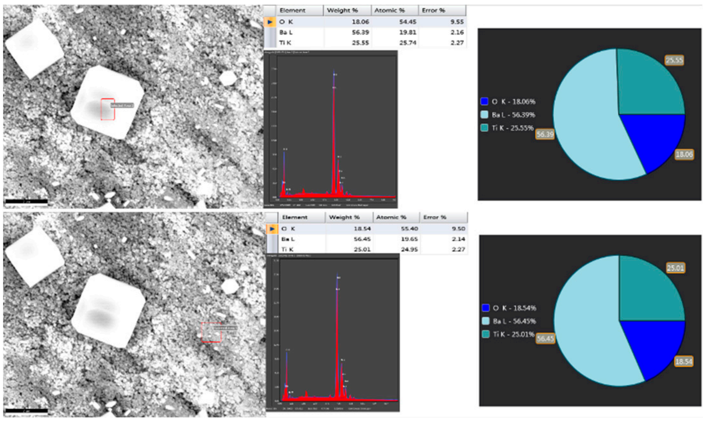

Figure S1. The EDS mapping.

The hJBMSCs were in spindle-shaped adherence. After 5–6 days, hJBMSCs grew rapidly and reached 80% confluence in a whirlpool (Figure S2a). The 3rd generations of hJBMSCs were induced to differentiate into osteoblasts and chondrocytes, which were identified by alizarin red staining (Figure S2b) and oil red O staining (Figure S2c). The results of flow cytometry screening of cell surface antigens in the third generation of hJBMSCs (Figure S2d) showed that CD90, CD73 and CD105 were positive, while CD34, CD45, HLA-DR and CD19 was negative, indicating no mixed hematopoietic stem cells in hJBMSCs.

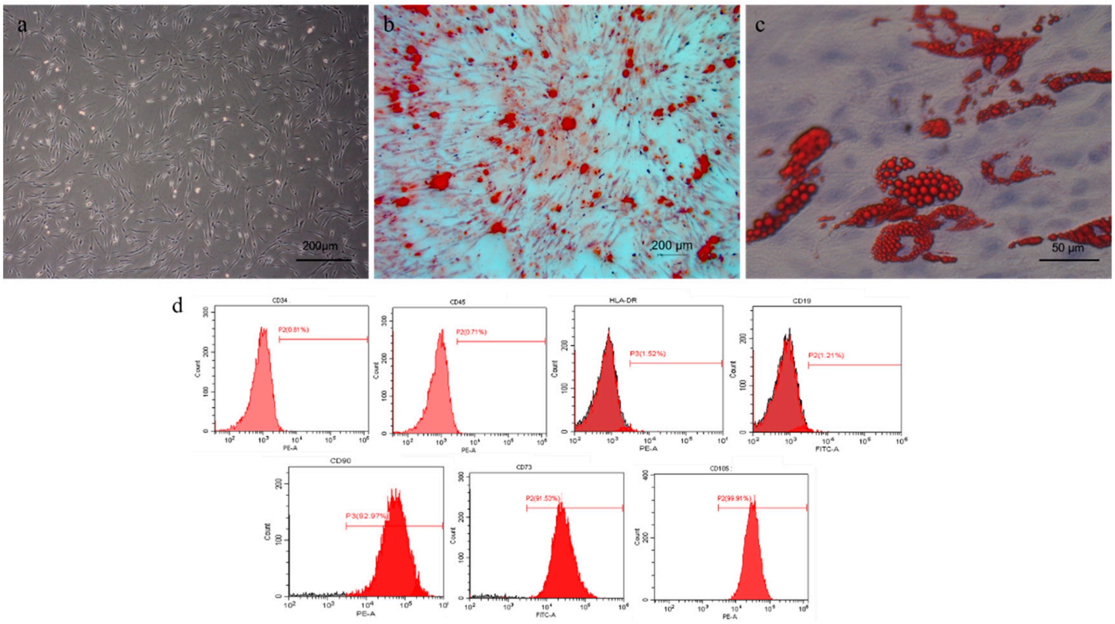

Figure S2. Identification of hJBMSCs. (**a**) hJBMSCs cultured for 5–6 days; (**b**) Alizarin red staining showed hJBMSCs had osteogenic differentiation ability; (**c**) oil red O staining showed hJBMSCs had lipogenic differentiation ability. (**d**) flow cytometry screening of cell surface antigens in the third generation of hJBMSCs showed that CD90, CD73 and CD105 were positive, while CD34, CD45, HLA-DR and CD19 was negative. (n = 3)
